# Identification of a novel fully human anti-toxic shock syndrome toxin (TSST)-1 single-chain variable fragment antibody averting TSST-1-induced mitogenesis and cytokine secretion

**DOI:** 10.1186/s12896-022-00760-8

**Published:** 2022-10-28

**Authors:** Mahdieh Soezi, Somayeh Piri-Gavgani, Mostafa Ghanei, Mir Davood Omrani, Behnoush Soltanmohammadi, Kamran Pooshang Bagheri, Reza Ahangari Cohan, Farzam Vaziri, Seyed Davar Siadat, Abolfazl Fateh, Shohreh Khatami, Masoumeh Azizi, Fatemeh Rahimi-Jamnani

**Affiliations:** 1 Department of Mycobacteriology and Pulmonary Research, Pasteur Institute of Iran, Tehran, Iran; 2 Microbiology Research Center, Pasteur Institute of Iran, Tehran, Iran; 3 Chemical Injuries Research Center, Systems Biology and Poisoning Institute, Baqiyatallah University of Medical Sciences, Tehran, Iran; 4 Department of Medical Genetics, School of Medicine, Shahid Beheshti University of Medical Sciences, Tehran, Iran; 5 Venom and Biotherapeutics Molecules Lab, Biotechnology Research Center, Pasteur Institute of Iran, Tehran, Iran; 6 Department of Nanobiotechnology, New Technologies Research Group, Pasteur Institute of Iran, Tehran, Iran; 7 Department of Biochemistry, Pasteur Institute of Iran, Tehran, Iran; 8 Molecular Medicine Department, Biotechnology Research Center, Pasteur Institute of Iran, Tehran, Iran

**Keywords:** *Staphylococcus aureus*, Superantigen, Toxic shock syndrome toxin-1, Autoimmune diseases, Toxic shock syndrome, Single-chain variable fragment

## Abstract

**Background:**

Staphylococcal superantigens are virulence factors that help the pathogen escape the immune system and develop an infection. Toxic shock syndrome toxin (TSST)-1 is one of the most studied superantigens whose role in toxic shock syndrome and some particular disorders have been demonstrated. Inhibiting TSST-1 production with antibiotics and targeting TSST-1 with monoclonal antibodies might be one of the best strategies to prevent TSST-1-induced cytokines storm followed by lethality.

**Results:**

A novel single-chain variable fragment (scFv), MS473, against TSST-1 was identified by selecting an scFv phage library on the TSST-1 protein. The MS473 scFv showed high affinity and specificity for TSST-1. Moreover, MS473 could significantly prevent TSST-1-induced mitogenicity (the IC_50_ value: 1.5 µM) and cytokine production.

**Conclusion:**

Using traditional antibiotics with an anti-TSST-1 scFv as a safe and effective agent leads to deleting the infection source and preventing the detrimental effects of the toxin disseminated into the whole body.

**Supplementary information:**

The online version contains supplementary material available at 10.1186/s12896-022-00760-8.

## Background

*Staphylococcus aureus* is a leading cause of nosocomial and community-acquired infections, such as skin and soft tissue infections, endocarditis, pneumonia, bacteremia, and toxic shock syndrome, which pose a serious challenge to the global health care system [1–3]. This pathogen is endowed with virulence factors with diverse functions, including cell wall-associated proteins, cytolysins, and superantigens, some of which function redundantly [1, 3, 4]. The staphylococcal superantigens such as staphylococcal enterotoxin serotype A (SEA), SEB, SEC, and toxic shock syndrome toxin (TSST)-1 interrupt the immune response against the pathogen via the antigen-independent activation of T cells and antigen-presenting cells (APCs) [5]. The simultaneous interaction of TSST-1 with the β-chain of the T-cell receptor (TCR) and the α-chain of the major histocompatibility complex (MHC) class II triggers a cascade of signaling pathways, leading to the hyperactivation of T cells and APCs and the release of large quantities of cytokines [5–7]. Nonspecific and excessive T cell activation by TSST-1 can result in a wide range of diseases, ranging from multi-organ failure in toxic shock syndrome to autoimmune diseases (e.g. rheumatoid arthritis and psoriasis) [5, 8–10].

There are several ways to control TSST-1 pathogenesis, including the use of antibiotics, killing the pathogen (e.g. vancomycin) or preventing protein synthesis (e.g. clindamycin and linezolid), and directly targeting the toxin [3, 8, 11–14]. Considering the emergence of antibiotic-resistant *S. aureus* strains and antibiotic-related side effects [1], targeting TSST-1, which can disseminate to various organs [15], in conjunction with antibiotics, may result in improved patients outcomes. Monoclonal antibodies (mAbs) are considered ideal biotherapeutics for neutralizing toxins [16, 17]. One of the sophisticated technologies used to generate mAbs is phage display, which has led to the development of antibodies with potential applications in the treatment of cancer, autoimmune disorders, and infectious diseases [17, 18]. It is noteworthy that Raxibacumab, one of the three anti-toxin mAbs approved by the Food and Drug Administration (FDA) [1, 16], has been developed by the phage display method [18].

There have been reports that anti-TSST mAbs neutralize the superantigenic effect of TSST-1 on human peripheral blood mononuclear cells (PBMCs) or murine spleen cells and protect animals against TSST-1-induced lethality [12, 13, 19]. In recent decades, antibody fragments such as single-chain variable fragments (scFvs) have gained increasing attention because of their outstanding binding properties, high tissue penetration, and appropriate clearance, making them effective anti-toxin agents [8, 20]. The scFv comprises a heavy-chain variable domain (VH) connected to a light-chain variable domain (VL) of an antibody by a peptide linker [1, 20]. Accordingly, Rukkawattanakul et al. developed three anti-TSST-1 scFvs, HuscFv35, HuscFv53, and HuscFv56, which inhibited massive T cell proliferation and proinflammatory cytokine production induced by TSST-1 [8].

In the present study, a human scFv phage display library was screened against the TSST-1 protein, leading to the identification of a novel scFv (MS473) with high affinity and specificity for TSST-1. The MS473 scFv was then demonstrated to inhibit TSST-1-induced mitogenesis and cytokine release in vitro.

## Results

### Selection of scFvs specific to TSST-1

**Fig. 1:**
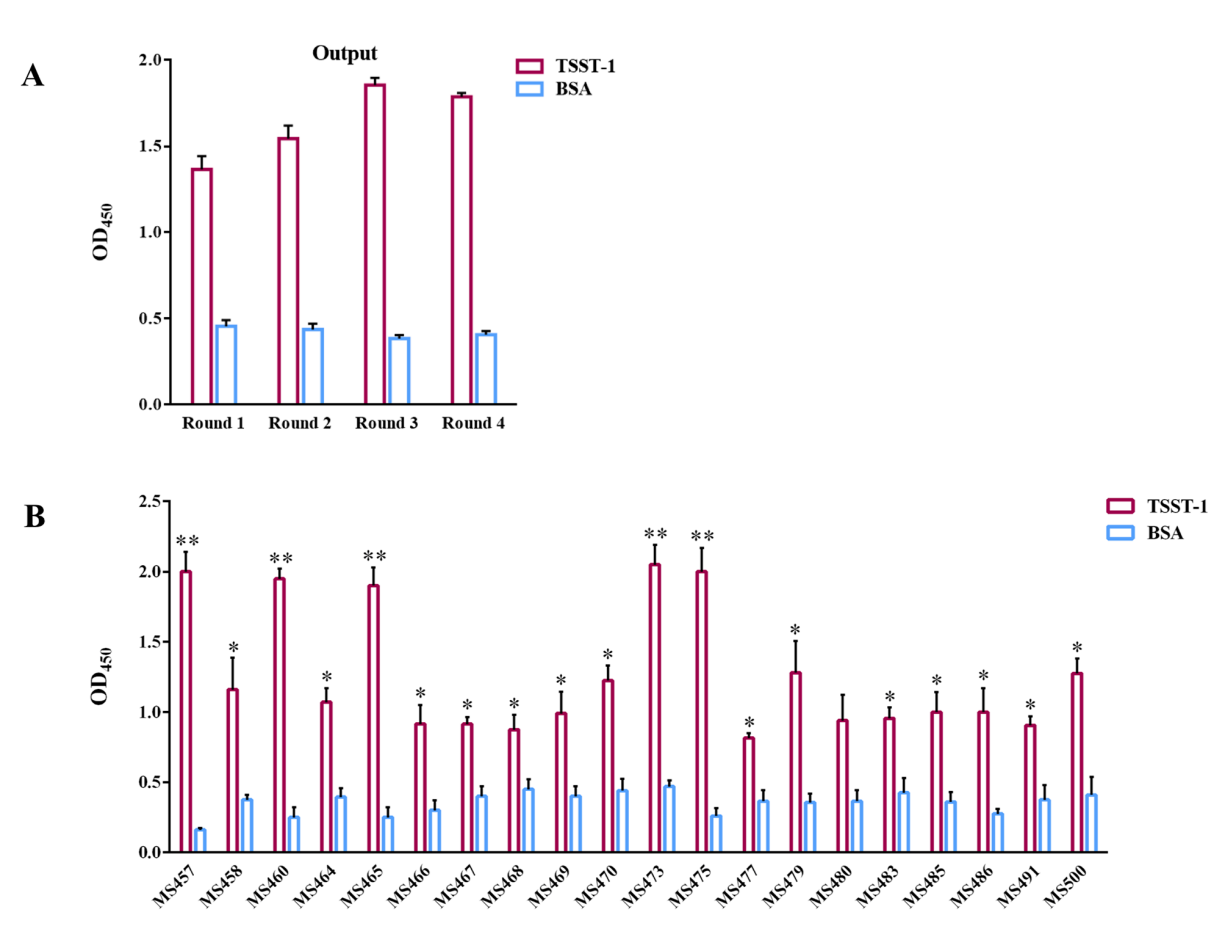
Screening of the scFv phage library on TSST-1. The TSST-1-binding of phages obtained from four rounds of biopanning on the TSST-1 protein was assessed by **(A)** polyclonal phage ELISA and **(B)** monoclonal phage ELISA. **(A)** The wells coated with TSST-1 or bovine serum albumin (BSA) were incubated with phages obtained from four rounds of biopanning, followed by horseradish peroxidase (HRP)-conjugated mouse anti-M13 antibody. The highest signal intensities were observed with output phages of the third and fourth round of biopanning on the TSST-1 protein compared to other output phages and the controls. **(B)** The binding of approximately 500 phage clones to TSST-1 was evaluated by ELISA. A total of 20 phage clones with potential binding to TSST-1 protein were identified, of which five, MS457, MS460, MS465, MS473, and MS475, had the highest binding signals. The samples were run in triplicate, and the results are expressed as mean ± standard error of the mean (SEM) of at least three individual experiments. Statistical comparisons were carried out with Student’s *t*-test. *P < 0.05, **P < 0.01

**Fig. 2:**
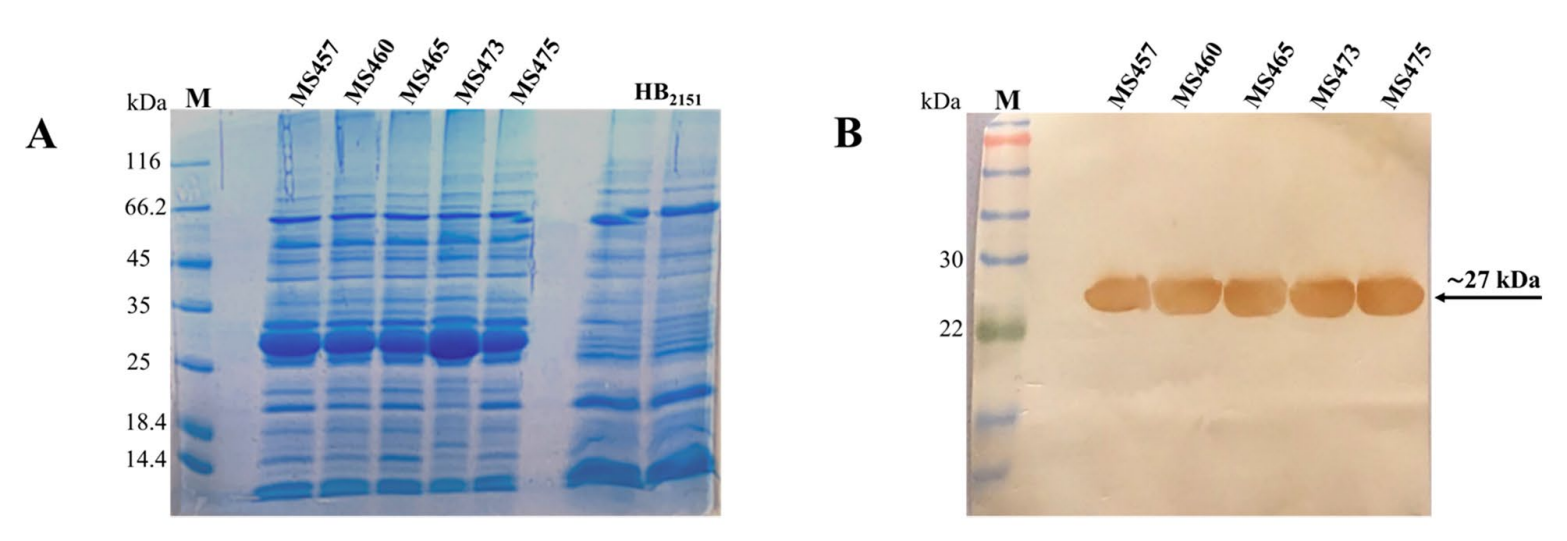
The expression of five scFvs in *E. coli* HB_2151_. The expression of five scFvs, MS457, MS460, MS465, MS473, and MS475, in *E. coli* HB_2151_, was evaluated by **(A)** SDS-PAGE and **(B)** western blot analysis. **(A)** The expression level of MS457, MS460, MS465, MS473, and MS475 (~ 27 kDa) in the periplasmic extract of *E.coli* HB_2151_ infected with the selected phagemids and the periplasmic extract of uninfected *E.coli* HB_2151_ (control) induced by 0.1 mM IPTG were assessed by an SDS-PAGE gel (12%). **(B)** A single band of the expected size (~ 27 kDa), related to the scFv, was detected by probing with mouse anti-human scFv polyclonal antibody, followed by goat anti-mouse IgG-horseradish peroxidase (HRP)-conjugated antibody. Lane M: pre-stained protein marker. The original images can be found in Additional file 1: Fig. S1

A human scFv phage display library was panned for four rounds against the TSST-1 protein. The number of input and output phages in each round of biopanning is presented in Table 1. The TSST-1-binding ability of phage populations obtained from each round of biopanning was examined by enzyme-linked immunosorbent assay (ELISA). As illustrated in Fig. 1A, the phages eluted from the third and fourth rounds of biopanning on the TSST-1 protein (output_3_ and output_4_) had the highest signal intensities compared to other output phages and the control protein. More than 500 *Escherichia coli* TG1 colonies infected with the outputs of the third and fourth rounds (output_3_ and output_4_) were evaluated for binding to TSST-1 by monoclonal phage ELISA. A total of 20 phage clones with potential binding to TSST-1 protein were identified, of which five, MS457, MS460, MS465, MS473, and MS475, had the highest binding signals (Fig. 1B). To generate soluble scFvs, *E. coli* HB_2151_ bacteria were infected with the selected scFv-phages. Next, the expression of MS457, MS460, MS465, MS473, and MS475 was induced with isopropyl β-d-1-thiogalactopyranoside (IPTG) and assessed by sodium dodecyl sulfate-polyacrylamide gel electrophoresis (SDS-PAGE) (Fig. 2 A). Western blot assay demonstrated a single band at approximately 27 kDa, corresponding to the scFv protein (Fig. 2B). Sequences analysis of five scFvs showed that MS457, MS460, MS465, MS473, and MS475 shared a common sequence. The MS473 scFv was selected For further characterizations due to its higher expression levels than the other four clones. The nucleotide sequence of MS473 was analyzed in the IMGT/V-QUEST database, and the results showed that the V-regions of VH and VL domains of MS473 were derived from the human germline IGHV1-46*01 F (or IGHV1-46*03 F) and IGKV1-39*01 F (or IGKV1D-39*01 F), respectively. Furthermore, the VH and VL domains of MS473 had a complementarity determining region 3 (CDR3) length of 14 and 9 amino acids, respectively (Additional file 2: Fig. S2).

**Table 1:** The number of input and output phages in each round of biopanning

Round	Input phage	Output phage	Ratio (Output / Input)
1	2 × 10^14^	3 × 10^7^	1.5 × 10^− 7^
2	5 × 10^14^	1 × 10^7^	0.2 × 10^− 7^
3	6 × 10^14^	8 × 10^7^	1.3 × 10^− 7^
4	8 × 10^14^	4 × 10^7^	0.5 × 10^− 7^

**Fig. 3:**
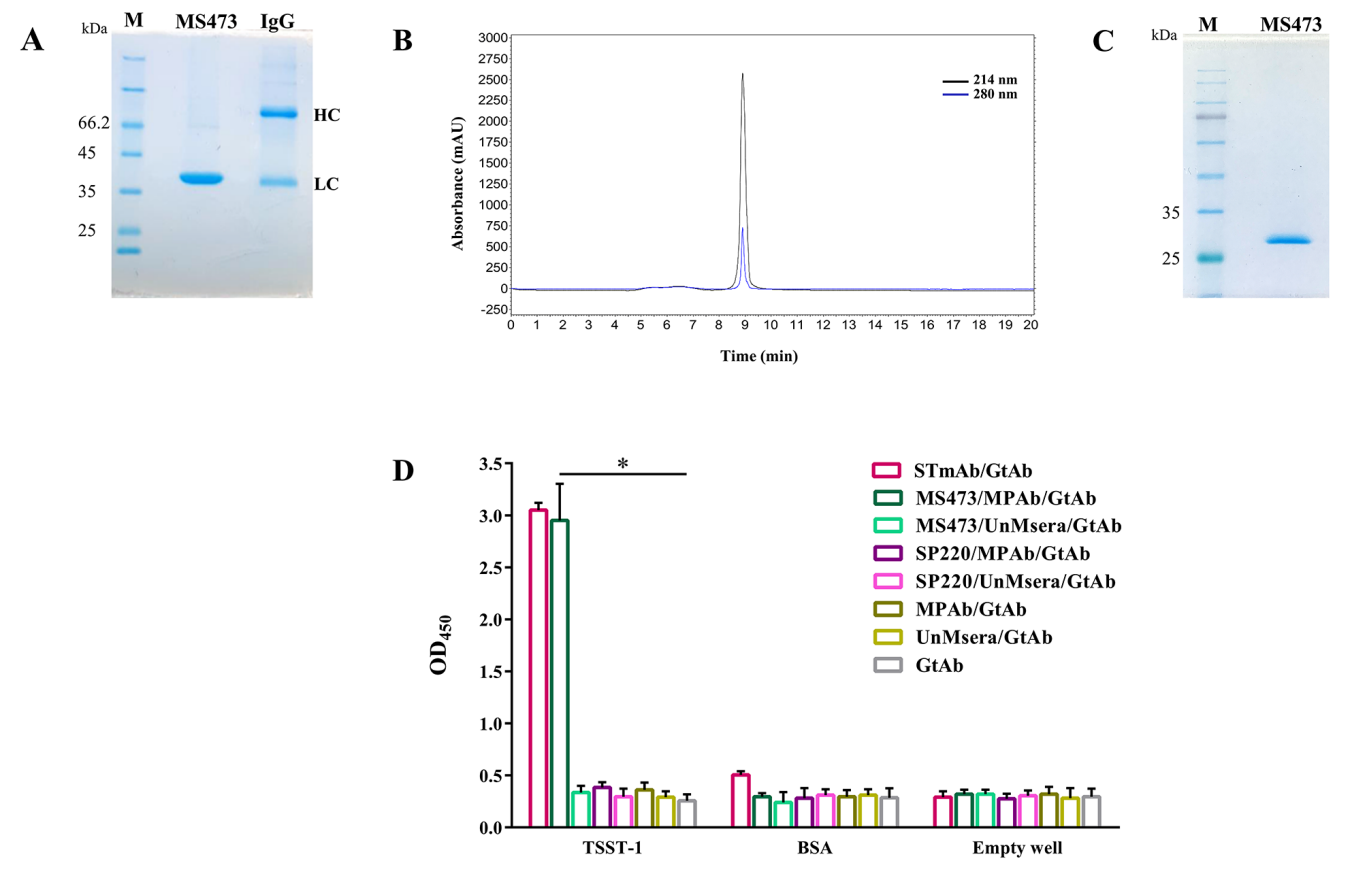
The significant binding of purified MS473 scFv to TSST-1. **(A)** Soluble MS473 scFv was purified by IMAC and analyzed by SDS-PAGE. A single protein band at approximately 27 kDa, corresponding to the expected molecular weight of the MS473 scFv, is seen on a 12% SDS-PAGE gel stained with Coomassie® blue. IgG: human immunoglobulin G, Lane M: unstained protein marker. The original image can be found in Additional file 3: Fig. S3. **(B)** Purified MS473 scFv was analyzed for aggregate content by SEC. The purified scFv was loaded onto the TSKgel G3000PWXL column, followed by elution with PBS. The eluted protein was detected by UV absorbance at 214 and 280 nm. **(C)** SDS-PAGE analysis of the SEC elution profile of MS473 scFv shows the presence of a highly pure band of 27 kDa, corresponding to the scFv protein. Lane M: pre-stained protein marker. The original image can be found in Additional file 4: Fig. S4. **(D)** The binding ability of purified MS473 scFv to TSST-1 was assessed by ELISA. The wells coated with TSST-1 or bovine serum albumin (BSA) (as control) were individually incubated with MS473 or SP220, followed by mouse anti-human scFv polyclonal antibody (MPAb) or unimmunized mouse sera (UnMsera; control). Empty wells were used as the control. Next, the wells were incubated with goat anti-mouse IgG-horseradish peroxidase (HRP)-conjugated antibody (GtAb), followed by the addition of the TMB solution. Other control groups include the TSST-1-coated wells, BSA-coated wells, and empty wells incubated directly with anti-staphylococcal TSST-1 mAb (STmAb) followed by GtAb; incubated directly with MPAb, followed by GtAb; incubated directly with UnMsera, followed by GtAb; or incubated directly with GtAb. The samples were run in triplicate, and the results are expressed as mean ± SEM of at least three individual experiments. Statistical comparisons were carried out with the one-way analysis of variance (ANOVA). *P < 0.05

The MS473 scFv was purified from the periplasmic extract by immobilized metal affinity chromatography (IMAC) on nickel-nitrilotriacetic acid (Ni-NTA) resin, followed by dialysis. The concentration of purified and dialyzed MS473 scFv was about 400 µg/ml, and the yield of purified MS473 was determined to be 1.2 mg. As shown in Fig. 3A, a single band at approximately 27 kDa, consistent with the expected size of the scFv, was observed on the SDS-PAGE gel (Fig. 3A). Moreover, purified MS473 scFv was subjected to size exclusion chromatography (SEC) to determine the presence of aggregates. As shown in Fig. 3B, a single sharp peak was detected on the size exclusion chromatogram of MS473. In addition, SDS-PAGE analysis of the concentrated elution fraction revealed a single band of approximately 27 kDa, corresponding to the scFv protein (Fig. 3C). The binding of purified MS473 scFv to TSST-1 was also examined by ELISA. As illustrated in Fig. 3D, the MS473 scFv and the commercial mouse anti-staphylococcal TSST-1 mAb showed strong binding to TSST-1 compared to the controls.

### Binding reactivity of MS473 to TSST-1

**Fig. 4:**
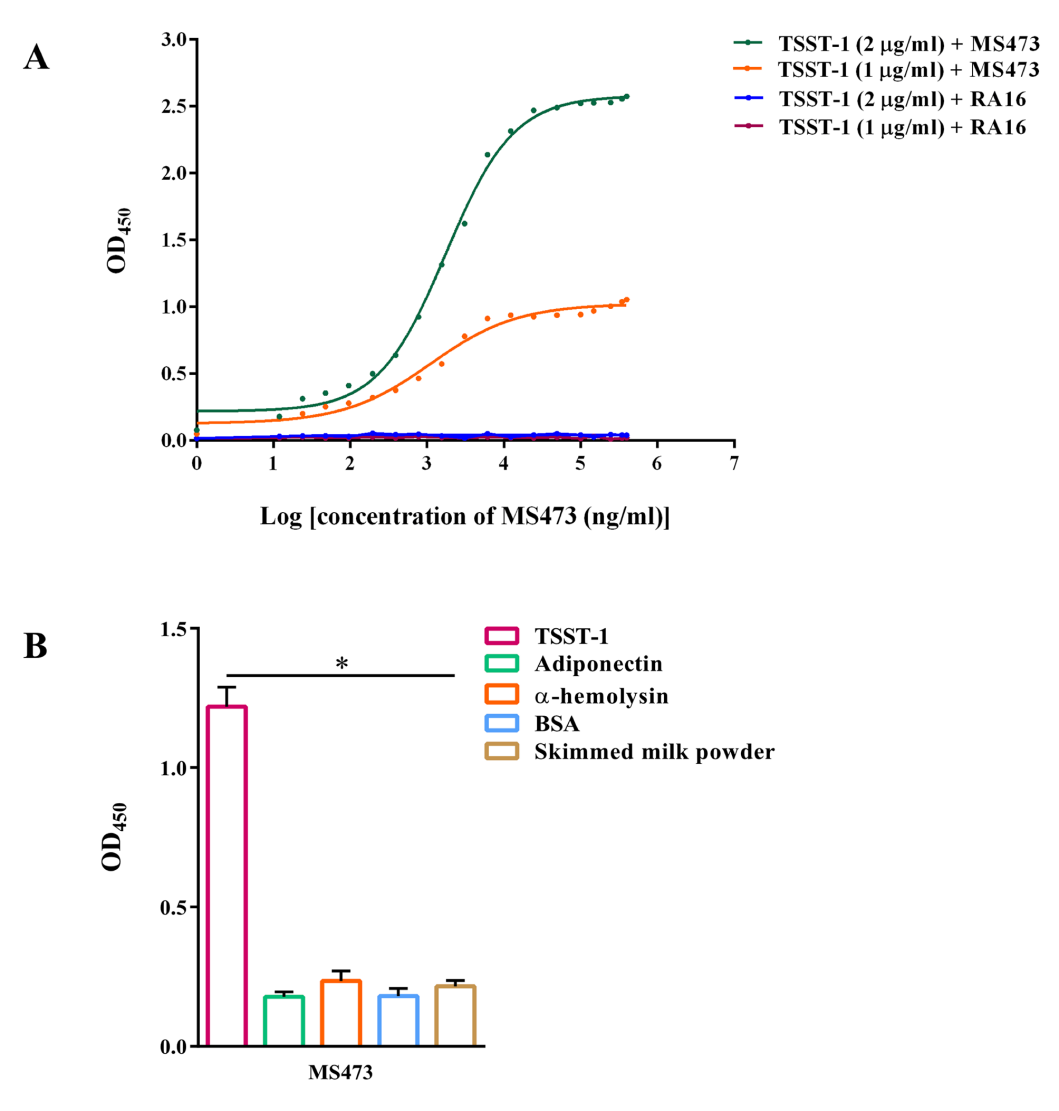
The binding affinity and specificity of MS473 to TSST-1. **(A)** The binding affinity of MS473 and RA16 (an scFv specific to IL-6) TSST-1 (1 and 2 µg/ml) was measured by non-competitive ELISA. **(B)** The specific binding of MS473 to TSST-1 was evaluated by ELISA. The wells coated with adiponectin, α-hemolysin, bovine serum albumin (BSA), skimmed milk powder, and TSST-1 were incubated with MS473, followed by mouse anti-human scFv polyclonal antibody and goat anti-mouse IgG-horseradish peroxidase (HRP)-conjugated antibody. The MS473 scFv exhibited significant binding to TSST-1 compared to the control proteins. The samples were run in triplicate, and the results are expressed as mean ± SEM of at least three individual experiments. Statistical comparisons were carried out with the one-way analysis of variance (ANOVA). *P < 0.05

The binding affinity of MS473 to TSST-1 was examined by incubating TSST-1 at concentrations of 1 and 2 µg/ml with different concentrations of scFv. The affinity constant (K_*aff*_) was calculated using the formula described previously by Beatty et al. [21]. Based on the results, the K_*aff*_ of the MS473 scFv was 0.4 ⋅ 10^9^ M^− 1^ (Fig. 4A).

The specific binding of MS473 to TSST-1 was appraised by ELISA using a group of proteins, including adiponectin, α-hemolysin, bovine serum albumin (BSA), and skimmed milk powder in addition to TSST-1. As shown in Fig. 4B, MS473 had significant binding to TSST-1 but not to other proteins tested.

### The interaction of MS473 with the TCR binding site on TSST-1

**Fig. 5:**
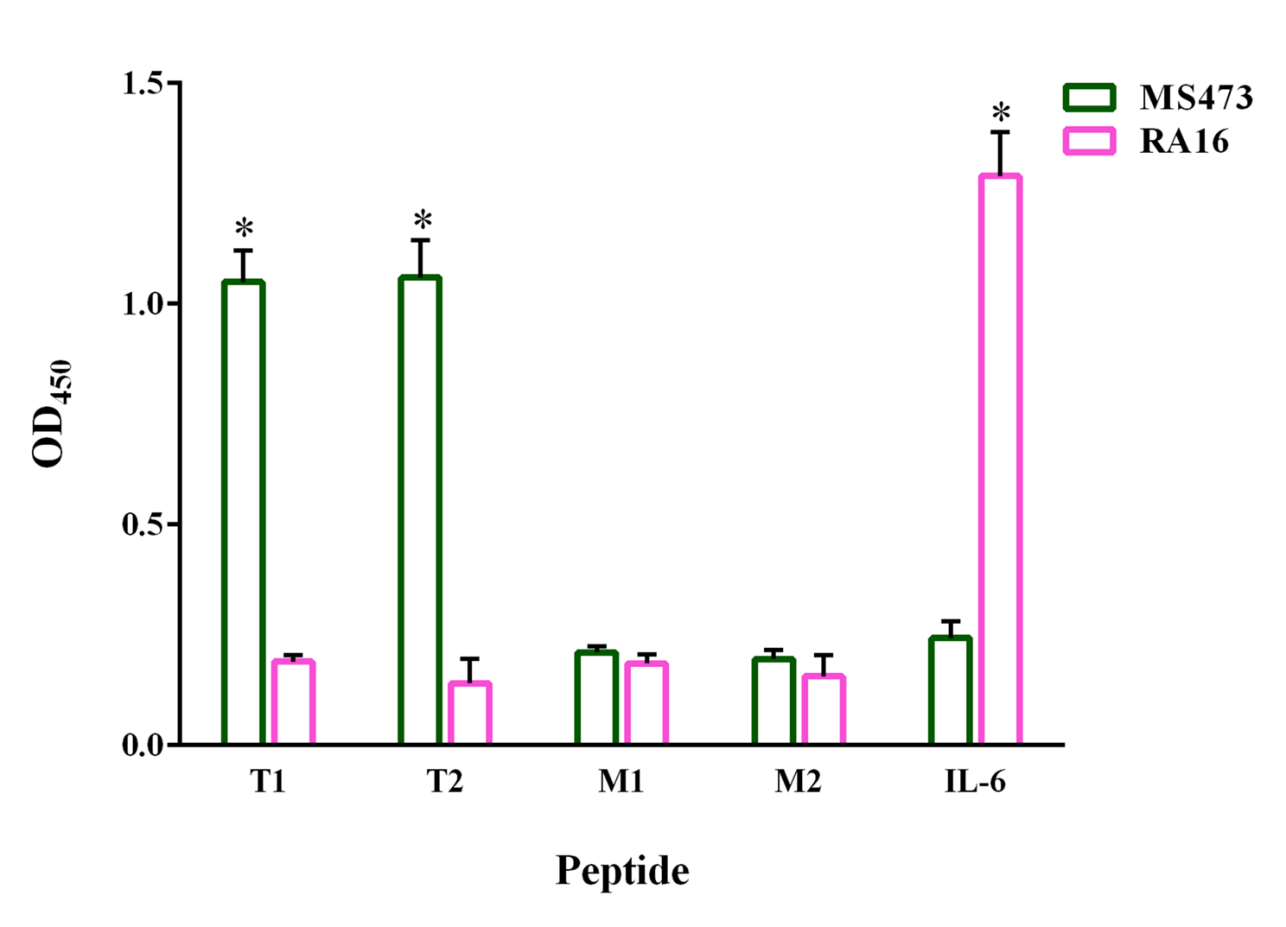
Reactivity of MS473 to TSST-1 peptides involved in the TCR binding. The MS473 scFv reacted significantly with the peptides T1 and T2, which are located in the region of TSST-1 that interacts with the human TCR Vβ-chain. There was no significant reaction between the MS473 scFv and peptides M1 and M2, both of which are located within the region of TSST-1 that interacts with the MHC class II. The RA16 scFv, a control scFv against IL-6, showed significant reactivity with the IL-6 peptide but not with the TSST-1 peptide. The samples were run in triplicate, and the results are expressed as mean ± SEM of at least three individual experiments. Statistical comparisons were carried out with Student’s *t*-test. *P < 0.01

An ELISA was used to assess the binding of MS473 to the peptides involved in the binding of TSST-1 to the TCR or MHC class II. As shown in Fig. 5, MS473 reacted with the peptides T1 and T2 involved in the binding of TSST-1 to the variable domain of the TCR β-chain (Vβ). In contrast, MS473 showed no significant binding activity for the peptides M1 and M2 involved in the interaction between TSST-1 and MHC class II. Moreover, no binding was observed between MS473 and the IL-6 peptide used as the control.

### Inhibition of TSST-1-induced mitogenesis and cytokine secretion by MS473

**Fig. 6:**
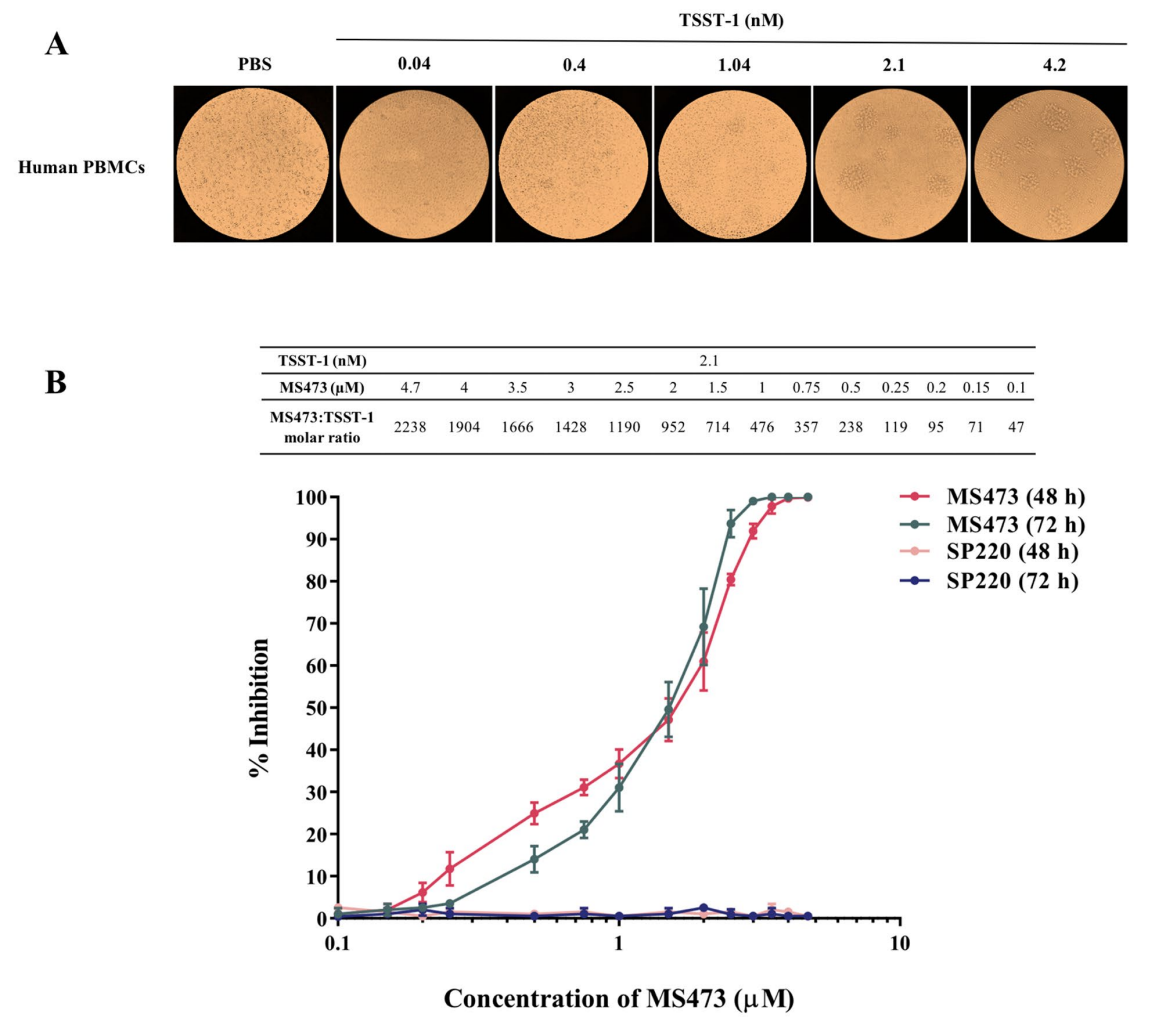
Inhibition of the mitogenic response of human peripheral blood mononuclear cells (PBMCs) to TSST-1 by MS473. **(A)** To determine the effective proliferative dose of TSST-1, fresh human PBMCs (~ 10^6^ cells/ml) were incubated with the TSST-1 protein at concentrations of 1, 10, 25, 50, and 100 ng/ml (0.04, 0.4, 1.04, 2.1, and 4.2 nM, respectively) for 48 h at 37 °C, 5% CO2. The cells incubated with PBS served as the control. The proliferation of PBMCs and the formation of cell clumps induced by TSST-1 were investigated using an inverted microscope **(B)** To determine the inhibitory activity of MS473 on TSST-1-induced mitogenesis, fresh human PBMCs (~ 10^6^ cells/ml) were incubated simultaneously with the TSST-1 protein (2.1 nM), and the serial dilutions of MS473 or SP220 (0.1–4.7 µM) for 48 and 72 h at 37 °C, 5% CO2. The cells incubated with the TSST-1 protein (2.1 nM) and PBS or PBS alone served as the controls. The MS473 scFv exhibited a significant inhibitory effect (the IC_50_ value: 1.5 µM) on the proliferation of PBMCs and decreased the number of colony-forming cells. The samples were run in triplicate, and the results are expressed as mean ± standard deviation (SD).

Human PBMCs were incubated with varying concentrations of TSST-1 (0.04, 0.4, 1.04, 2.1, and 4.2 nM). The results indicated that TSST-1 had a significant mitogenic effect at concentrations greater than 2.1 nM (Fig. 6A). To examine the TSST-1-neutralizing activity of MS473, human PBMCs were incubated concurrently with TSST-1 (2.1 nM) and a series of concentrations of MS473 or SP220 (an unrelated scFv) (0.1–4.7 µM) for 48 and 72 h. The viability of PBMCs was appraised by an inverted microscope and the MTT assay. Based on the results, MS473 could significantly inhibit the proliferative effect of TSST-1 in a dose-dependent manner after 48 and 72 h of incubation (Additional file 5: Fig. S5) (Fig. 6). The MS473 scFv showed a complete inhibitory activity on TSST-1-induced proliferation of PBMCs at concentrations more than 3.5 µM (∼ 100% inhibition). The 50% inhibitory concentration (IC_50_) of MS473 was determined to be 1.5 µM. In contrast, the SP220 scFv at the highest concentration (4.7 µM) displayed no inhibitory effect on TSST-1-induced mitogenesis (Fig. 6B).

**Fig. 7:**
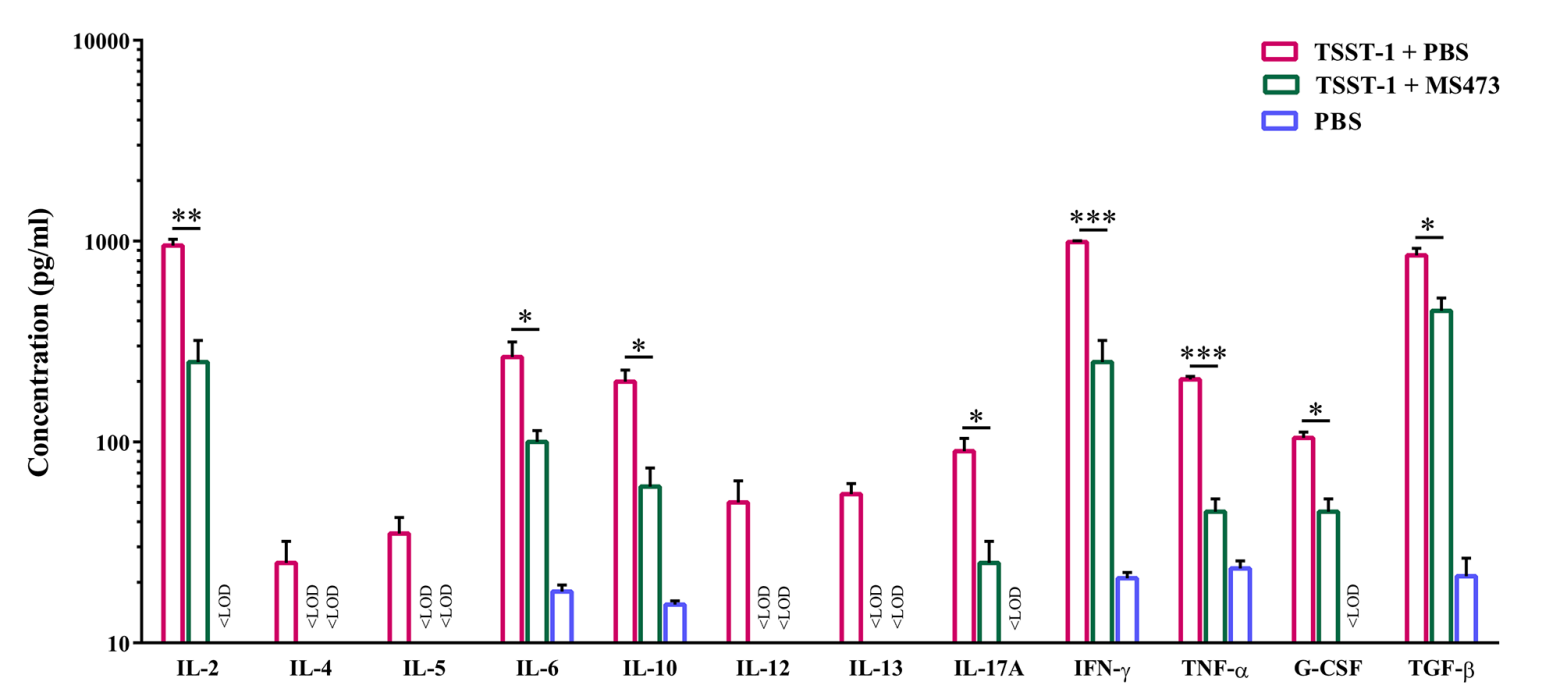
Inhibition effects of MS473 on the production of cytokines from human peripheral blood mononuclear cells (PBMCs) stimulated with TSST-1. To assess the inhibition effect of MS473 on the TSST-1-induced cytokine secretion, fresh human PBMCs (~ 10^6^ cells/ml) were incubated simultaneously with the TSST-1 protein (2.1 nM) and MS473 (3 µM) for 24 h at 37 °C, 5% CO2. The cells treated with PBS or the TSST-1 protein (2.1 nM) and PBS served as the controls. The concentration of human interleukin 2 (IL)-2, IL-4, IL-5, IL-6, IL-10, IL-12, IL-13, IL-17 A, interferon (IFN)-γ, tumor necrosis factor (TNF)-α, G-CSF, and transforming growth factor (TGF)-β in the culture supernatant of PBMCs (stimulated and unstimulated) were calculated using the standard curve provided in a Multi-Analyte ELISArray Kit (Qiagen). The concentration of cytokines (pg/ml) is represented on a logarithmic scale. The concentrations of IL-4, IL-5, IL-12, and IL-13 in the culture supernatant of PBMCs treated with TSST1 and MS473 (or PBS) were less than the limit of detection (LOD). Moreover, the concentrations of IL-2, IL-17 A, and G-CSF in the culture supernatant of PBMCs treated with PBS were less than the LOD. The samples were run in triplicate, and the results are expressed as mean ± SD. *P < 0.05, **P = 0.01, ***P < 0.01

Furthermore, treatment of human PBMCs with TSST-1 (2.1 nM) and MS473 (3 µM) led to the decreased secretion of interleukin (IL)-2, IL-4, IL-5, IL-6, IL-10, IL-12, IL-13, IL-17 A, interferon (IFN)-γ, tumor necrosis factor (TNF)-α, granulocyte colony-stimulating factor (G-CSF), and transforming growth factor (TGF)-β compared to the control group (human PBMCs treated with TSST-1 and phosphate buffer saline [PBS]) (Fig. 7).

## Discussion

The potential involvement of TSST-1 in a group of complicated disorders, including atherosclerosis [22] and autoimmune diseases (e.g. rheumatoid arthritis, psoriasis) [3, 9, 23], Kawasaki syndrome, and sudden infant death syndrome [9, 23], as well as its prominent role in the toxic shock syndrome [5, 24], underscore the necessity of finding functional agents targeting TSST-1. The history of applying antibodies to neutralize toxins and three FDA-approved mAbs in the market (Bezlotoxumab, Obiltoxaximab, and Raxibacumab) [16] make mAbs substantial anti-TSST-1 agents. In this regard, two neutralizing mAbs targeting TSST-1 have been developed [12, 13]. TSST-1-neutralizing mAbs blocked toxin activity by interfering in the binding of TSST-1 to the TCR or MHC class II in an Fc-independent manner [12, 13]. Therefore, antibody fragments such as scFv antibodies may be good alternatives due to their small size, high tissue penetration, low immunogenicity, and low-cost and easy production [1, 8, 20]. To isolate TSST-1-specific scFvs, we enriched a fully human scFv phage library against the TSST-1 protein in this study. A novel scFv, MS473, was identified, and its binding reactivity was assessed by ELISA. The MS473 scFv showed high-affinity binding to TSST-1 (K_*aff*_ = 0.4 ⋅ 10^9^ M^− 1^). Moreover, MS473 bound specifically to TSST-1 but not to the control proteins, including adiponectin, α-hemolysin, BSA, and skimmed milk powder.

TSST-1 exerts its mitogenic activity by bridging the TCR on T cells and MHC class II on APCs in an antigen-independent manner [5]. Of note, Huseby et al. showed that treating the mononuclear cells with TSST-1 (1 µg/well) resulted in the formation of lymphocytes colonies as brown clumps [25]. In this respect, we treated human PBMCs with TSST-1 (2.1 nM) and a range of concentrations of MS473 (0.1–4.7 µM) to determine whether the binding of the scFv to TSST-1 led to the inhibition of the proliferation of PBMCs stimulated with TSST-1. Based on the results, MS473 had a significant inhibition effect on the proliferation of PBMCs and reduced the number of colony-forming cells (the IC_50_ value: 1.5 µM). The effect of MS473 on the secretion of a group of cytokines from PBMCs induced by TSST-1 was also evaluated by a Multi-Analyte ELISArray Kit. The data indicated that MS473 could decrease the release of cytokines, including IL-2, IL-4, IL-5, IL-6, IL-10, IL-12, IL-13, IL-17 A, IFN-γ, TNF-α, G-CSF, and TGF-β from TSST-1-stimulated PBMCs in comparison with PBMCs treated with TSST-1 alone. The human PBMC population consists of various cells, including B cells, dendritic cells, monocytes, natural killer cells, CD8^+^ T cytotoxic cells, and CD4^+^ T helper (h) cells (e.g. naïve T cells, Th1, Th2, Th17, and T regulatory) [26, 27]. Therefore, TSST-1-mediated activation of APCs and CD4^+^ T cell subsets in PBMCs might lead to the secretion of a cocktail of cytokines ranging from pro-inflammatory to inhibitory cytokines [5, 23, 28–30]. However, the neutralization of TSST-1 by MS473 could reduce TSST-1-induced cytokine production in PBMCs. In this regard, Bonventre et al. developed a murine IgG mAb against TSST-1, designated MAb 8-5-7, blocking TSST-1-induced mitogenesis in murine spleen cells, inhibiting IL-1 production from human PBMCs stimulated with TSST-1, and protecting the rabbits against TSST-1-induced lethality [12]. In another study, Kum et al. reported that MAb5, a mouse IgG1 mAb, had a significant inhibitory effect on TSST-1-induced mitogenicity (the IC_50_ value: 1 nM), and IL-1β, IL-6, and TNF-α secretion in human PBMCs [13]. Based on their results, MAb5 targeted TSST-1 residues 51YYSPAF56, involved in MHC class II binding of TSST-1. Moreover, they showed that MAb5 prolonged the survival rate in the subcutaneous TSST-1 infusion rabbit model and the D-galactosamine sensitized murine model of lethal shock [13]. In the study by Pang et al., MAb5 had a neutralization effect on SEB-induced superantigenic activity in human PBMCs (e.g. inhibition of SEB-induced mitogenicity and TNF-α release) [31]. However, its neutralization activity was 1000-fold lower than against TSST-1 [31]. In a similar study, Kum and Chow [32] showed that MAb5 inhibited SEA-induced mitogenicity and TNF-α production in human PBMCs, and protected mice against SEA-induced lethality. Moreover, their results indicated that MAb5 had a lower inhibitory potency on SEA than on TSST-1, due to its lower binding affinity to SEA (780 nM^− 1^) than TSST-1 (0.9 nM^− 1^). Consistent with our study, Rukkawattanakul et al. isolated three anti-TSST-1 scFv antibodies (HuscFv35, HuscFv53, and HuscFv56) from a human scFv phage library [8]. To assess the neutralization activity of HuscFv35, HuscFv53, and HuscFv56, human PBMCs (5 ⋅ 10^4^ cells/well) were incubated with the TSST-1 protein (1000 ng/ml) and the HuscFv (4 µg/well**)** for 24 h. Based on the results, HuscFv35 and HuscFv56 exhibited more inhibitory effects on the proliferation of T cells and production of IL-1β, IL-6, and TNF**-**α in PBMCs induced by TSST-1 than that of HuscFv53 [8]. Based on the in-silico docking analysis, HuscFv35 disturbed the binding of TSST-1 to the TCR, leading to decreased cytokine secretion induced by TSST-1. They predicted that HuscFv53 interacted with TSST-1 residues (Arg68 or Tyr80) involved in the binding to the HLA-DR2. Moreover, their data showed that HuscFv56 contacted multiple critical residues on TSST-1, impacting the mitogenicity and superantigenicity of TSST-1 [8]. We examined the interaction site between MS473 and TSST-1 using an ELISA-peptide assay to determine how MS473 inhibited the mitogenic activity of TSST-1. The results exhibited that MS473 reacted significantly with the peptides T1 and T2, which corresponded to the residues 10–26 and 110–145 of TSST-1, involved in the binding to the human TCR Vβ-chain [33–36], but not with the peptides M1 or M2, involved in the binding to the MHC class II [13, 34, 37]. It has been reported that the residues Gly16, Trp116, Glu132, His135, Gln136, and Gln139 played a critical role in interacting between TSST-1 and the human TCR Vβ2.1-chain [35]. Therefore, we speculated that the MS473 scFv inhibited the TSST-1 activity by likely interfering with its binding to the human TCR Vβ-chain.

There was no data about the IC_50_ value or TSST-1-binding affinity of HuscFv35, HuscFv53, and HuscFv56 developed by Rukkawattanakul et al. [8]. In contrast, the MS473 scFv displayed high-affinity binding to TSST-1 (K_*aff*_: 0.4 ⋅ 10^9^ M^− 1^), leading to the inhibition of PBMCs proliferation (the IC_50_ value: 1.5 µM) and a significant reduction in the secretion of a group of inflammatory and anti-inflammatory cytokines in PBMCs induced with 2.1 nM TSST-1.

## Conclusion

We developed a high affinity fully human scFv, MS473, with a specific binding ability to TSST-1. The MS473 scFv could significantly affect TSST-1-induced mitogenesis and decrease the release of an array of cytokines from human PBMCs stimulated with TSST-1. Moreover, the protective activity of MS473 against TSST-1-induced lethality is also being evaluated in a D-galactosamine–sensitized mouse model of lethal shock. Considering the role of TSST-1 in toxic shock syndrome and some disorders with unknown causes, conventional antibiotics combined with a neutralizing scFv with substantial pharmacokinetic and pharmacodynamic profiles may be effective in treating patients with complex conditions.

## Methods

### Isolation of phages expressing scFvs specific to TSST-1

To isolate TSST-1-specific phages, a large fully human scFv phage display library with total diversity of 2 × 10^10^ (Creative Biolabs) was enriched against the TSST-1 protein as described previously with some modifications [1, 38]. Briefly, a 96-well MaxiSorp plate (Nunc, Roskilde, Denmark) was coated with 100 µl of the TSST-1 protein (2 µg/ml in NaHCO3) (Sigma-Aldrich, St. Louis, MO, USA) overnight at 4 °C. Following blocking the plate (15 mg/ml BSA in PBS containing 0.1% tween-20 [PBS-T]), pre-blocked phages (~ 10^12^ plaque-forming unit/ml) amplified from the scFv phage library were added to the wells, and incubation was done for 90 min at room temperature (RT). Following several times washing with PBS-T, bound phages were eluted (output) and amplified (input) for further biopanning rounds. This procedure was repeated for four rounds. Washing steps were increased from round one to round four (10, 15, 20, and 25 times, respectively). The output/input ratio of each round was determined to evaluate the enrichment efficiency [39, 40]. To verify the success of the biopanning process, polyclonal phage ELISA was carried out as described previously [38, 39, 41–44]. In brief, a 96-well MaxiSorp plate was coated with 100 µl of the TSST-1 protein (2 µg/ml) or BSA (2 µg/ml) (Merck, Darmstadt, Germany) overnight at 4 °C. The wells were blocked, followed by the incubation with output phages obtained from the first to the fourth round of biopanning (output_1_-output_4_) for 60 min at RT. After multiple washing steps with PBS-T, horseradish peroxidase (HRP)-conjugated mouse anti-M13 antibody (1:2000 dilution in blocking buffer) (Santa Cruz Biotechnology, INC.) was added to the wells, followed by the incubation for 60 min at RT. Next, the wells were washed several times with PBS-T, and the signals were generated by adding 3,3′,5,5′-Tetramethylbenzidine (TMB) (Thermo Scientific, MA, US). The reactions were stopped with sulfuric acid (1 M) (Merck), and the absorbance at 450 nm was measured using a microplate reader (Epoch, BioTek, USA).

Based on the data obtained from polyclonal phage ELISA, output phages of the third and fourth rounds of biopanning (output_3_ and output_4_), showing the highest signal intensity compared to the control, were further assessed by monoclonal phage ELISA [38]. Briefly, *E. coli* TG1 bacteria were infected with output phages (output_3_ and output_4_) and cultured on lysogeny broth (LB) agar (Merck) plates with 150 µg/ml ampicillin (Sigma-Aldrich). After incubation overnight at 37 °C, the colonies were picked up randomly, and phage amplification was done as described previously [1]. The binding ability of phages to the TSST-1 protein was investigated by ELISA, as mentioned above in polyclonal phage ELISA.

### Expression

Five phage clones, MS457, MS460, MS465, MS473, and MS475, which showed the highest binding to the TSST-1 protein compared to the control in monoclonal phage ELISA, were selected for more evaluations. To produce soluble scFv antibodies, the non-suppressor *E. coli* strain, HB_2151_, was infected with the selected scFv-phages and cultured on LB agar plates containing ampicillin, followed by incubation overnight at 37 °C [1, 45]. The single colonies were picked up and cultured in 100 ml terrific broth expression medium containing 100 µg/ml ampicillin. After adding IPTG (0.1 mM) (Thermo Scientific) and incubating overnight at 24 °C, the cultures were centrifuged, and the pellets were incubated with the lysis buffer for 60 min at RT [45, 46]. Next, the existing level of scFvs in the periplasmic fraction of *E. coli* HB_2151_ bacteria carrying the phagemids (MS457, MS460, MS465, MS473, or MS475) was investigated by a 12% SDS-PAGE gel, followed by western blot analysis. After electrophoresis, the proteins were transferred from the SDS-PAGE gel (12%) onto the polyvinylidene fluoride (PVDF) membrane (GE Healthcare, Little Chalfont, UK). The blocked membrane was incubated with mouse anti-human scFv polyclonal antibody (1:200 dilution) for 60 min at RT [1]. After several washing steps with tris-buffered saline (TBS) with 0.05% tween-20 (TBS-T) and TBS, the membrane was incubated with goat anti-mouse IgG-HRP-conjugated antibody (1:2000 dilution) (Santa Cruz) for 60 min at RT, followed by several washing steps and addition of 3,3′-diaminobenzidine substrate (DAB) (Sigma-Aldrich) and hydrogen peroxide (H_2_O_2_) (Merck).

### Sequencing

The phagemid DNA of clones MS457, MS460, MS465, MS473, and MS475, was purified using the High Pure Plasmid Isolation Kit (Roche, Mannheim, Germany), based on the manufacturer’s recommendation. For sequencing, the forward primer, 5’- CTA TGA CCA TGA TTA CGA ATT TCT A -3’, was used. The nucleotide sequences of five scFvs were appraised using the Gene Runner program (version 6.0). Furthermore, the amino acid sequences of V-regions of MS473 were analyzed by the IMGT/V-QUEST tool (http://www.imgt.org/IMGT_vquest/analysis) [1].

### Assessment of the binding ability of the purified scFv to TSST-1

The soluble scFv, MS473, was purified using a Ni-NTA column (Qiagen, Hilden, Germany), according to the manufacturer’s instructions. The bound proteins were eluted with 200 mM imidazole (Merck). Next, all the eluted fractions were pooled and then placed in a dialysis bag (cut off 14 kDa, Sigma-Aldrich), according to the manufacturer’s instructions. The concentration of purified and dialyzed scFv (MS473) was measured via the Bradford assay. The purity of the scFv was analyzed by a 12% SDS-PAGE gel. Moreover, SEC (TSKgel G3000PWXL column) (Tosoh Bioscience, Tokyo, Japan) was used to determine the extent of aggregation. The concentrated elution fraction was assessed by SDS-PAGE.

The binding ability of purified scFv to TSST-1 was determined by ELISA as described previously with some modifications [38]. Briefly, a 96-well MaxiSorp plate was coated with 100 µl of the TSST-1 protein (2 µg/ml) or BSA (2 µg/ml) (as the control). Next, the wells were blocked and then incubated with MS473 or SP220 (an scFv against staphylococcal α-hemolysin) (400 µg/ml) for 60 min at RT. After several washing steps, a mouse anti-human scFv polyclonal antibody was added to the wells, and incubation was done for 60 min at RT. The wells were washed several times with PBS-T and PBS, and a goat anti-mouse IgG-HRP-conjugated antibody was added to the wells, followed by incubation for 60 min at RT. Moreover, the TSST-1-coated wells that were incubated with a commercial mouse anti-staphylococcal TSST-1 mAb (1/1000 dilution) (Santa Cruz), followed by a goat anti-mouse IgG-HRP-conjugated antibody were used as the positive control. After multiple washing steps, the TMB substrate solution was added to the wells, and the color reactions were stopped with sulfuric acid. A microplate reader determined the absorbance at 450 nm.

### Affinity determination

The binding affinity of MS473 to TSST-1 was determined as described previously [21, 38, 47, 48]. In brief, a 96-well MaxiSorp plate was coated with 100 µl of the TSST-1 protein (1 and 2 µg/ml). After blocking, the wells were incubated with serial dilution of the MS473 scFv or the RA16 scFv (an anti-IL-6 scFv) (0.02–450 µg/ml) for 60 min at RT. After several washing steps, a mouse anti-human scFv polyclonal antibody was added to the wells, and incubation was done for 60 min at RT. Next, the wells were washed multiple times and incubated with goat anti-mouse IgG-HRP-conjugated antibody for 60 min at RT. After adding the TMB substrate solution, the color development was stopped with sulfuric acid. The absorbance was read at 450 nm. The K_*aff*_ of MS473 to the TSST-1 protein was measured using the following formula:

$$n{\rm{ }} = {\rm{ }}{\left[ {Ag} \right]_t}/{\rm{ }}{\left[ {Ag^{\prime}} \right]_t}$$

$${K_{aff}}_ = n{\rm{ }}-{\rm{ }}1{\rm{ }}/{\rm{ }}2{\rm{ }}\left( {n{\rm{ }}{{\left[ {scFv^{\prime}} \right]}_t}-{\rm{ }}{{\left[ {scFv} \right]}_t}} \right)$$

Where [Ag]_t_ is the total concentration of TSST-1; [scFv]_t_ and [scFv’]_t_ are the total concentration of MS473 at OD-50 and OD-50’ for the wells coated with the TSST-1 protein at 2 and 1 µg/ml, respectively.

### Specificity

The binding specificity of MS473 to TSST-1 was assayed by ELISA as described previously [38]. In brief, a 96-well MaxiSorp plate was coated with 100 µl of the TSST-1 protein (2 µg/ml), the adiponectin protein (2 µg/ml) (R&D Systems, Minnesota, US), the α-hemolysin protein (2 µg/ml) (Merck, Calbiochem, Germany), BSA (2 µg/ml), and skimmed milk powder (1 mg/ml). After blocking, the wells were individually incubated with MS473 (200 µg/ml), followed by several washing steps with PBS-T and PBS. Next, a mouse anti-human scFv polyclonal antibody was added to the wells, followed by a goat anti-mouse IgG-HRP-conjugated antibody. After multiple washing steps, the TMB substrate solution was added to the wells, and the color reactions were stopped with sulfuric acid. A microplate reader determined the absorbance at 450 nm.

### Evaluation of the interaction site between MS473 and TSST-1

An ELISA-peptide assay was conducted as previously described with some modifications [49–51]. Initially, the TSST-1 peptides (residues 10–24 [peptide T1] and residues 110–145 [peptide T2], involved in the binding to the human TCR Vβ-chain [33–36], and residues 27–60 [peptide M1] and residues 72–86 [peptide M2], involved in the binding to the HLA-DR1 molecule) [13, 34, 37], and a human interleukin-6 peptide (residues 28–62; an unrelated peptide) were synthesized with a GGK linker at the C-terminus for biotinylation and purified to a purity of 95% by high-pressure liquid chromatography (HPLC) (Biomatik, Ontario, Canada). For ELISA, 96-well streptavidin-coated plates (Pierce, Rockford, IL) were coated with 100 pM biotin-labeled peptides and incubated overnight at 4 °C, followed by several washing steps with TBS-T containing 0.1% BSA. Next, the coated peptides were incubated with 4 µM MS473 or RA16 (an anti-IL-6 scFv) for 60 min at RT. After several washing steps, the wells were incubated with anti-human scFv polyclonal antibody, followed by goat anti-mouse IgG-HRP-conjugated antibody. By adding the TMB substrate solution, the color development was stopped with sulfuric acid, and the absorbance at 450 nm was measured using a microplate reader.

### Assessment of the inhibition ability of MS473 on the TSST-1-induced mitogenesis and cytokine release in human PBMCs

The neutralizing activity of MS473 against the superantigenic activity of TSST-1 was examined on human PBMCs isolated from the whole blood of two healthy donors (men, 50 and 45 years) as described previously with some modifications [8, 12, 13, 31, 52, 53]. To determine the effective proliferative dose of TSST-1, fresh human PBMCs (~ 10^6^ cells/ml) in RPMI 1640 medium (Gibco; Grand Island, NY, USA) with L-glutamine (2 mM) (Gibco) supplemented with heat-inactivated fetal bovine serum (10%) (Gibco) were seeded in 96-well round-bottom tissue culture plates (JET BIOFIL, Guangzhou, China) and were incubated with the TSST-1 protein at concentrations of 1, 10, 25, 50, and 100 ng/ml (0.04, 0.4, 1.04, 2.1, and 4.2 nM, respectively) for 48 h at 37 °C, 5% CO2. The proliferation of PBMCs and the formation of cell clumps induced by TSST-1 were investigated using an inverted microscope (BEL, Monza, Italy) [25].

Next, the cells were incubated concurrently with the TSST-1 protein (2.1 nM) and the scFv (MS473 or SP220 [an unrelated scFv]) at concentrations ranging from 0.1 to 4.7 µM for 48 and 72 h at 37 °C, 5% CO2. Moreover, the cells incubated with PBS or the TSST-1 protein (2.1 nM) and PBS were used as the controls. The inhibitory effect of MS473 on the proliferation of PBMCs stimulated with TSST-1 was assessed by an inverted microscope and the MTT assay as described previously [54]. The percentage of inhibition was calculated using the formula as follows:

$$\% \,Inhibition\, = \,100\, - \,\left[ {\frac{{({A_{scFv}}\, - \,{A_{PBS}})}}{{({A_{Toxin}}\, - \,{A_{PBS}})}}\, \times \,100} \right]$$

Where A_scFv_ is the absorbance (at 450 nm) of PBMCs treated with TSST-1 and scFv; A_PBS_ is the absorbance of PBMCs treated with PBS; A_Toxin_ is the absorbance of PBMCs treated with TSST-1 and PBS.

Moreover, the culture supernatant of PBMCs treated simultaneously with the TSST-1 protein (2.1 nM) and MS473 (3 µM) were collected after 24 h of incubation at 37 °C, 5% CO2, followed by centrifugation at 1000 g for 5 min. The cells treated with PBS or the TSST-1 protein (2.1 nM) and PBS served as the controls. The concentrations of human IL-2, IL-4, IL-5, IL-6, IL-10, IL-12, IL-13, IL-17 A, IFN-γ, TNF-α, G-CSF, and TGF-β in the culture supernatant of PBMCs (stimulated and unstimulated) were calculated using the standard curve provided in a Multi-Analyte ELISArray Kit (Qiagen), according to the manufacturer’s instructions. Experimental procedures with human blood were approved by the Ethics Committee of the Pasteur Institute of Iran and were done in accordance with the Helsinki Declaration. The participant provided written informed consent before enrollment.

## Statistical analyses

The one-way analysis of variance (ANOVA) and Student’s *t*-test were carried out in GraphPad Prism version v.6.0.7. The *P*-value < 0.05 was considered significant.

## Electronic supplementary material

Below is the link to the electronic supplementary material.

Supplementary Material 1

Supplementary Material 2

Supplementary Material 3

Supplementary Material 4

Supplementary Material 5

## Data Availability

All data generated or analyzed during this study are included in the manuscript and supplementary files.
