# Supplementary material for: Identification of a novel fully human anti-toxic shock syndrome toxin (TSST)-1 single-chain variable fragment antibody averting TSST-1-induced mitogenesis and cytokine secretion
Source: BMC Biotechnol. 2022 Oct 28;22:31. doi: 10.1186/s12896-022-00760-8 (PMC9617332; doi:10.1186/s12896-022-00760-8)
Supplement: Supplementary file 1 — Supplementary Material 1 [file 12896_2022_760_MOESM1_ESM.docx]

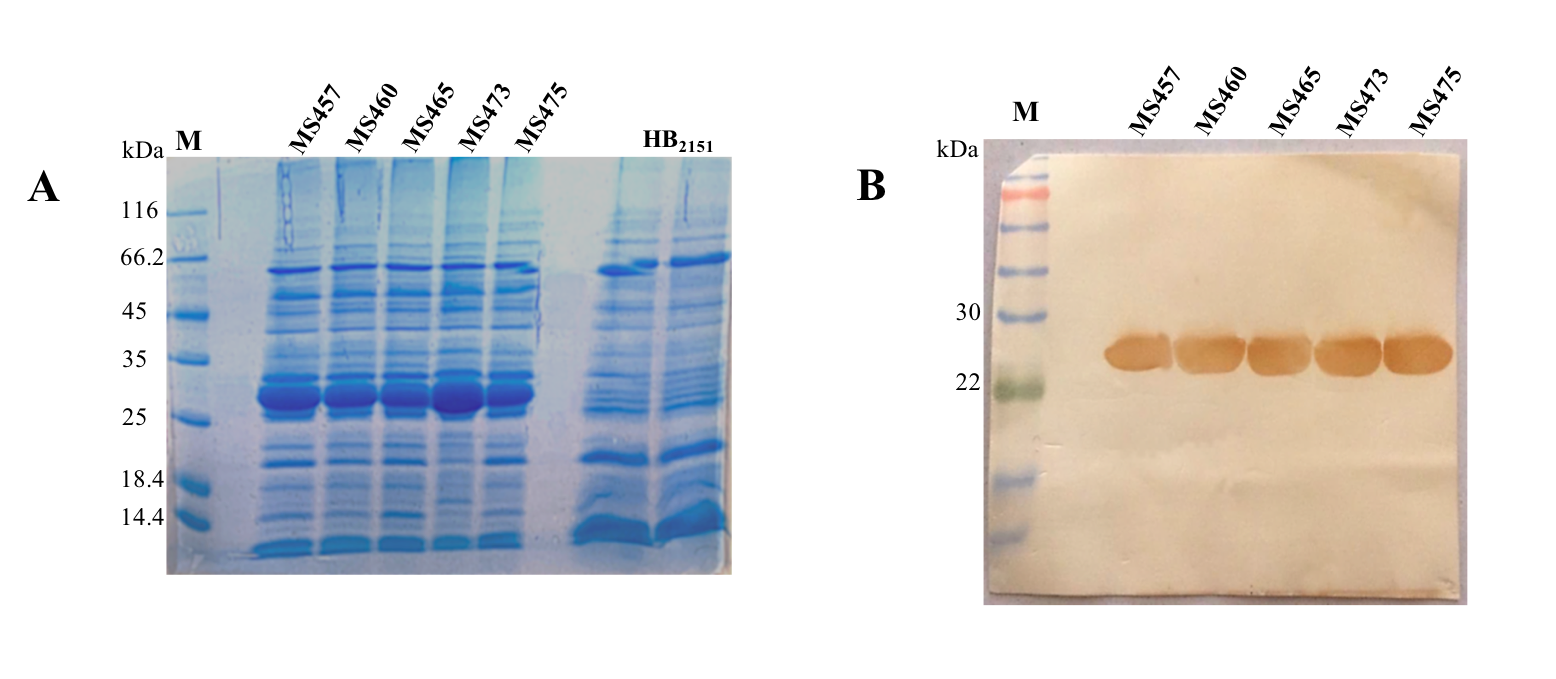


**Additional file 1: Supplementary Fig. S1.** The expression of five soluble scFvs in *E. coli* HB_2151_. The expression of five scFvs, MS457, MS460, MS465, MS473, and MS475, in *E. coli* HB_2151_, was evaluated by **(A)** SDS-PAGE and **(B)** western blot analysis. **(A)** The expression level of MS457, MS460, MS465, MS473, and MS475 (~ 27 kDa) in the periplasmic extract of *E.coli* HB_2151_ infected with the selected phagemids and the periplasmic extract of uninfected *E.coli* HB_2151_ (control) induced by 0.1 mM IPTG were assessed by an SDS-PAGE gel (12 %). Lane M: Unstained protein marker. **(B)** A single band of the expected size (~ 27 kDa), related to the scFv, was detected by probing with mouse anti-human scFv polyclonal antibody, followed by goat anti-mouse IgG-horseradish peroxidase (HRP)-conjugated antibody. Lane M: pre-stained protein marker.
