# Supplementary material for: Identification of a novel fully human anti-toxic shock syndrome toxin (TSST)-1 single-chain variable fragment antibody averting TSST-1-induced mitogenesis and cytokine secretion
Source: BMC Biotechnol. 2022 Oct 28;22:31. doi: 10.1186/s12896-022-00760-8 (PMC9617332; doi:10.1186/s12896-022-00760-8)
Supplement: Supplementary file 2 — Supplementary Material 2 [file 12896_2022_760_MOESM2_ESM.docx]

FR1

FR3

**CDR2**

**CDR1**

FR2

**//**QVQLVQSGAEVKKPGASVKVSCKAS**//**GYTIFYYQ**//**MHWVRQAPGQGLEWMGI**//**ISNPGSTG**//**SYAQKFLGRITMTNETSTSTAYNDL

FR1

**CDR3**

FR4

SSLRSEDTAYVYC**//**ARELGKSPAVAFDI**//**WGQATLATASSLE**//**GGGGSGGGGSGGGAS**//**DIQMTQSPSSLSASVGDRVTITCRVS

FR4

**CDR3**

**CDR1**

FR3

FR2

**CDR2**

**//**QIGSNT**//**LNWYHQKPGKVPKLLIS**//**SGAV**//**SLQSGAPSRFSGSGSGTDFTLTISSLQPEDFATYYC**//**KRISLWVKT**//**FGGGTKVDIK

RAVAHHHHHH

**Additional file 2: Supplementary Fig. S2.** The amino acid sequence of MS473**.**
