# Supplementary material for: Identification of a novel fully human anti-toxic shock syndrome toxin (TSST)-1 single-chain variable fragment antibody averting TSST-1-induced mitogenesis and cytokine secretion
Source: BMC Biotechnol. 2022 Oct 28;22:31. doi: 10.1186/s12896-022-00760-8 (PMC9617332; doi:10.1186/s12896-022-00760-8)
Supplement: Supplementary file 3 — Supplementary Material 3 [file 12896_2022_760_MOESM3_ESM.docx]

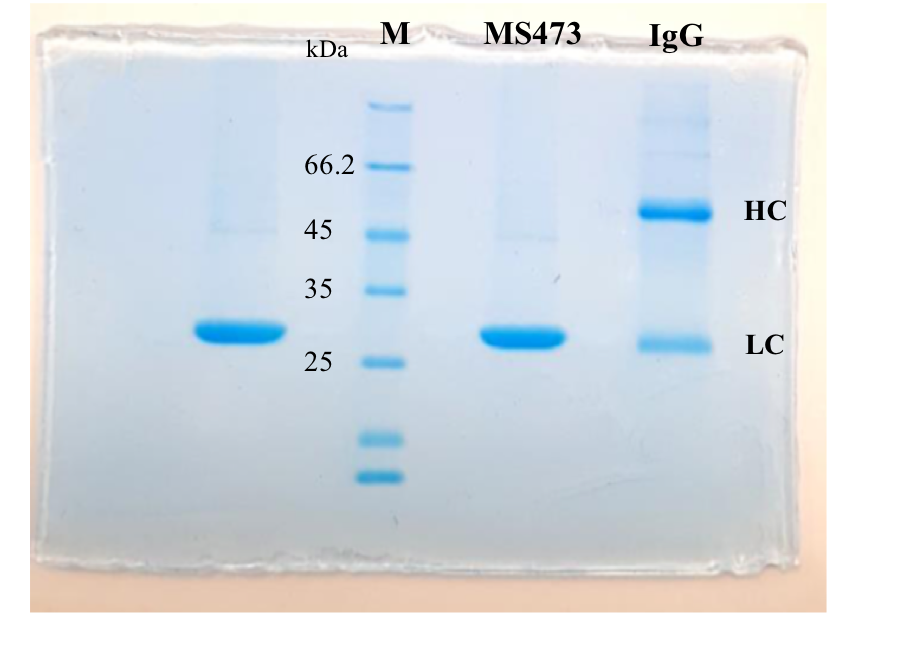


**Additional file 3: Supplementary Fig. S3.** The purity of MS473 was evaluated by SDS-PAGE. A single protein band at approximately 27 kDa related to the purified scFv is seen on a 12% SDS-PAGE gel stained with Coomassie® blue. IgG: human immunoglobulin G, Lane M: unstained protein marker. The red box on the original SDS-PAGE denoted the region cropped for the Fig. 3A shown in the manuscript.
