# Supplementary material for: Identification of a novel fully human anti-toxic shock syndrome toxin (TSST)-1 single-chain variable fragment antibody averting TSST-1-induced mitogenesis and cytokine secretion
Source: BMC Biotechnol. 2022 Oct 28;22:31. doi: 10.1186/s12896-022-00760-8 (PMC9617332; doi:10.1186/s12896-022-00760-8)
Supplement: Supplementary file 4 — Supplementary Material 4 [file 12896_2022_760_MOESM4_ESM.docx]

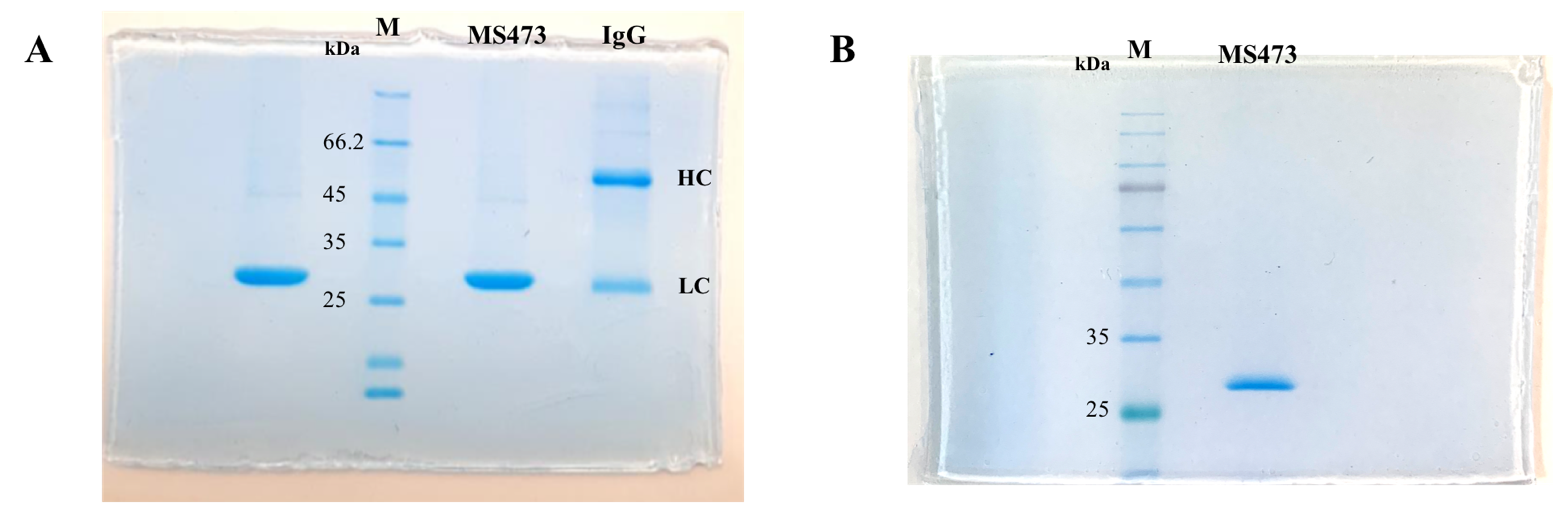


**Additional file 4: Supplementary Fig. S4.** SDS-PAGE analysis of the SEC elution profile of MS473 scFv shows the presence of a highly pure band of 27 kDa, corresponding to the scFv protein. Lane M: pre-stained protein marker. The red box on the original SDS-PAGE denoted the region cropped for the Fig. 3C shown in the manuscript.
