# Supplementary material for: Identification of a novel fully human anti-toxic shock syndrome toxin (TSST)-1 single-chain variable fragment antibody averting TSST-1-induced mitogenesis and cytokine secretion
Source: BMC Biotechnol. 2022 Oct 28;22:31. doi: 10.1186/s12896-022-00760-8 (PMC9617332; doi:10.1186/s12896-022-00760-8)
Supplement: Supplementary file 5 — Supplementary Material 5 [file 12896_2022_760_MOESM5_ESM.docx]

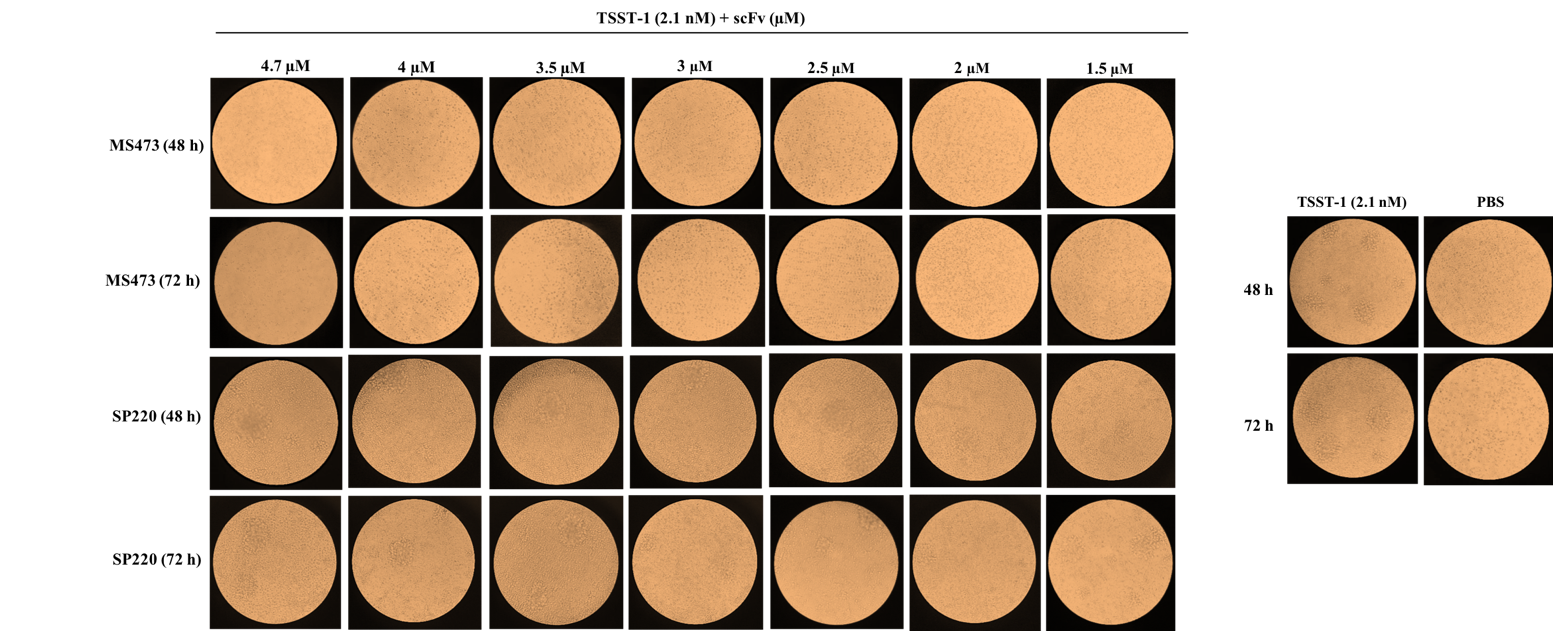


**Additional file 5: Supplementary Fig. S5.** Inhibition of the mitogenic response of human peripheral blood mononuclear cells (PBMCs) to TSST-1 by MS473. Fresh human PBMCs (~ 10^6^ cells/ml) were incubated simultaneously with the TSST-1 protein (2.1 nM), and the serial dilutions of MS473 or SP220 for 48 and 72 hours at 37°C, 5% CO2. The cells incubated with the TSST-1 protein (2.1 nM) and PBS or PBS alone served as the controls. The proliferation of PBMCs and the formation of cell clumps induced by TSST-1 were investigated using an inverted microscope.
